# Formation of few-electron triple quantum dots in ZnO heterostructures

**DOI:** 10.1038/s41598-025-20567-9

**Published:** 2025-10-21

**Authors:** Koichi Baba, Kosuke Noro, Yusuke Kozuka, Takeshi Kumasaka, Motoya Shinozaki, Masashi Kawasaki, Tomohiro Otsuka

**Affiliations:** 1 Research Institute of Electrical Communication, Tohoku University, 2-1-1 Katahira, Aoba-ku, Sendai, 980-8577 Japan; 2 Graduate School of Engineering, Tohoku University, 6-6 Aramaki Aza Aoba, Aoba-ku, Sendai, 980-0845 Japan; 3 Research Center for Materials Nanoarchitechtonics (MANA), National Institute for Material Science (NIMS), 1-2-1 Sengen, Tsukuba, 305-0047 Japan; 4 WPI Advanced Institute for Materials Research, Tohoku University, 2-1-1 Katahira, Sendai, 980-8577 Japan; 5 Department of Applied Physics and Quantum-Phase Electronics Center (QPEC), University of Tokyo, 7-3-1 Hongo, Bunkyo-ku, Tokyo, 113-8656 Japan; 6 RIKEN Center for Emergent Matter Science (CEMS), 2-1 Hirosawa, Wako, 351-0198 Japan; 7 Center for Science and Innovation in Spintronics, Tohoku University, 2-1-1 Katahira, Aoba-ku, Sendai, 980-8577 Japan

**Keywords:** Semiconductor quantum dot, Zinc oxide, A few-electron states, Quantum cellular automata effect, Electronic devices, Electronic devices, Quantum information

## Abstract

In recent years, advancements in semiconductor manufacturing technology have enabled the formation of high-quality, high-mobility two-dimensional electron gases in zinc oxide (ZnO) heterostructures, making the electrostatic formation of quantum dots possible. ZnO, with its low natural abundance of isotopes possessing nuclear spin and its direct band gap, is considered a potentially suitable material for quantum bit applications. In this study, we achieve the formation of triple quantum dots and the realization of a few-electron state in ZnO heterostructure devices. We also confirm that by varying the gate voltage between the quantum dots, it is possible to control the interdot coupling. Additionally, we observe a tunneling phenomenon called a quantum cellular automata effect, where multiple electrons move simultaneously, which is not seen in single or double quantum dots, due to Coulomb interactions. Our results demonstrate that ZnO nanostructures have reached a level where they can function as controllable multiple quantum dot systems.

## Introduction

Advancements in semiconductor microfabrication technology have made it possible to create nanoscale fine structures. A representative example of this is semiconductor quantum dots (QDs), which can confine electrons within nanoscale regions, enabling the observation of quantum mechanical properties^1–3^. Research is underway to utilize the electron spins confined in quantum dots as quantum bits (qubits)^4–8^, the fundamental units of quantum information systems. Semiconductor quantum dots have the intrinsic potential for large-scale integration, and the spins of electrons confined within the dots exhibit long coherence times^9–14^, allowing for highly precise qubit operations. This has raised expectations for realizing quantum computers using semiconductor qubits^15,16^.

**Fig. 1:**
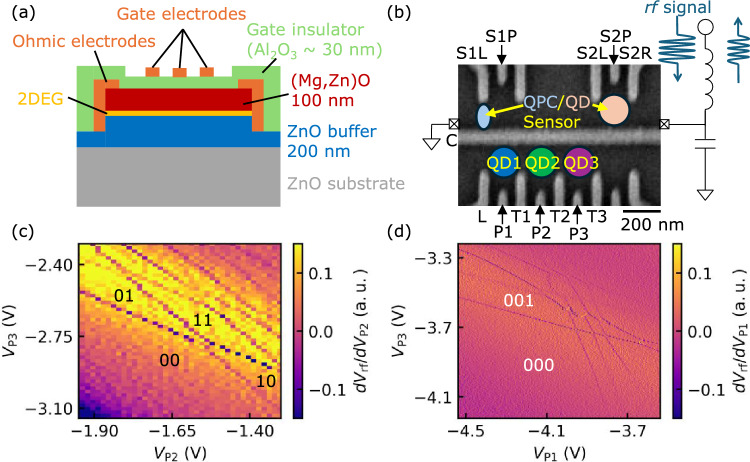
(**a**) A cross-sectional schematic view of the ZnO heterostructure device used in the experiment. (**b**) Scanning electron microscope image of the device. The positions of the formed quantum dots are also drawn. (**c**) Charge stability diagram of double quantum dots. The number of electrons in each quantum dot is denoted as (n$_{1}$ n$_{2}$). Gate voltage settings used for this measurement are $V_{\mathrm{{C}}} =-3.80$ V, $V_{\mathrm{{L}}} = -1.15$ V, $V_{\mathrm{{T1}}} = -2.00$ V, $V_{\mathrm{{T2}}} = -3.10$ V, $V_{\mathrm{{T3}}} = -3.40$ V, $V_{\mathrm{{S2L}}} = -3.34$ V, $V_{\mathrm{{S2P}}} = -3.25$ V, and $V_{\mathrm{{S2R}}} = -3.38$ V. (**d**) Charge stability diagram of triple quantum dots. The number of electrons in each quantum dot is shown as (n$_{1}$ n$_{2}$ n$_{3}$). Gate voltage settings used for this measurement are $V_{\mathrm{{C}}} =-3.80$ V, $V_{\mathrm{{L}}} = -1.33$ V, $V_{\mathrm{{T1}}} = -1.71$ V, $V_{\mathrm{{T2}}} = -3.20$ V, $V_{\mathrm{{T3}}} = -3.60$ V, $V_{\mathrm{{P2}}} = -1.93$ V, $V_{\mathrm{{S2L}}} = -3.34$ V, $V_{\mathrm{{S2P}}} = -3.34$ V, and $V_{\mathrm{{S2R}}} = -3.38$ V.

Recently, high-quality and high-mobility two-dimensional electron gases (2DEGs) have been realized in zinc oxide (ZnO) heterostructure devices^17^. This has enabled the observation of quantum phenomena such as the quantum Hall effect^17–20^ and quantum point contacts (QPCs)^21^ on ZnO devices, as well as the electrostatic formation of quantum dots^22,23^. ZnO, being a direct band gap semiconductor, exhibits strong coupling with light^24^, and its low abundance of isotopes with nuclear spin is expected to result in a long electron spin coherence time^25,26^. These characteristics make ZnO a promising material for applications such as quantum bits.

To realize quantum computers, it is necessary to integrate quantum bits, and semiconductor qubits offer the advantage of utilizing existing integration technologies. Semiconductor spin qubits have already demonstrated high-precision quantum state manipulation^12,27–30^, and their scalability has been advanced through the development of multi-quantum dot devices^14,31–36^. However, research has thus far been limited to the formation of single and double quantum dots in ZnO heterostructures^22,23^, making the development of multiple quantum dots, as well as the demonstration of elementary qubit operations, the next challenges for utilizing the unique properties of ZnO in quantum applications.

The scaling up of quantum dot systems contributes to quantum information processing with large-scale qubits while also providing platforms to explore fundamental quantum physics phenomena. Quantum computers require integrating a large number of qubits, making the development of scalable multi-quantum dot architectures essential. Beyond applications, multi-quantum dot systems with three or more quantum dots exhibit unique physical phenomena not observable in single or double dots^37–40^. Among these phenomena, the quantum cellular automata (QCA) effect^41–44^, a tunneling phenomenon where multiple electrons move simultaneously through Coulomb interactions, is of special interest. This effect has been studied for information transfer between qubits or as a mechanism for quantum information processing itself. Experimental measurements of the real-time dynamics of this effect^45^ have provided fundamental insights into electron transport mechanisms in multi-quantum dot systems.

To utilize the advantages of ZnO, such as potential long spin coherence times, establishing multi-quantum dots with precise control capabilities is essential. The formation of few-electron states and the gate voltage control of interdot couplings are fundamental requirements for both qubit operations and the investigation of quantum phenomena. Precise gate control enables the systematic exploration of quantum states and transport phenomena by allowing us to adjust energy levels and tunnel barriers. These technological developments are the key to opening the potential of ZnO in both quantum information applications and fundamental quantum understanding.

In this study, we fabricate a multi-quantum dot sample using a ZnO heterostructure and obtain the charge stability diagram of a triple quantum dot. Furthermore, we demonstrate the formation of a few-electron state in the triple quantum dot and show that control between quantum dots is possible by varying the gate voltage. Additionally, we observe a correlated tunneling phenomenon in ZnO, where multiple electrons move simultaneously through Coulomb interactions, a phenomenon not observed in single or double quantum dots.

## Results and discussion

### Device structure

Figure 1a shows a cross-sectional view of the ZnO heterostructure device used in this experiment. From top to bottom, it consists of a stacked structure of an Al$_{2}\textrm{O}_{3}$ insulating film, (Mg, Zn)O, a ZnO buffer, and a ZnO substrate. Additionally, a 2DEG is formed at the interface between the (Mg, Zn)O layer and the ZnO buffer layer.

In this study, radio-frequency (rf) reflectometry is used to obtain charge stability diagrams. RF reflectometry^23,46–48^ utilizes quantum point contacts or quantum dots as charge sensors, reading the resistance of the charge sensor as the intensity of rf reflections. Compared to transmission current measurements, it offers the advantages of faster and lower-noise measurements. Figure 1b shows a scanning electron micrograph of the fabricated device. Triple quantum dots are formed in the lower part, and the charge state is probed by a sensor quantum dot on the upper side. The Target QD is formed by L, P1, P2, P3, T1, T2, and T3. Note that the two gates in the lower-right corner are not used in this experiment. A quantum dot is formed by S2R, S2P, and S2L, while a quantum point contact by S1R and S1P. These QD and QPC serve as charge sensors, constructing a resonance circuit as shown in Fig. 1b. The QPC sensor is employed for the measurement of the QCA charge states, as described later. A resonant frequency of 174 MHz is used for the QD sensor. All measurements are conducted in a dilution refrigerator with a base temperature of 60 mK.

Figure 1c shows a charge stability diagram obtained by using P2, P3, T1, T2, and T3 to form double quantum dots. The reflected rf signal $V_{\textrm{rf}}$ from the sensor quantum dot is measured while varying $V_{\textrm{P2}}$ and $V_{\textrm{P3}}$ under the gate voltage conditions given in the figure caption. The existence of two different slopes of the charge transition lines confirms the formation of double quantum dots. In Fig. 1c, we find that charge transition lines disappear over a wide range in the lower left region, where no electrons are present in each quantum dot. This result indicates that it is possible to realize and observe a few-electron state in our device.

### Formation of multiple quantum dots

Next, we form the triple quantum dots. Figure 1d shows a charge stability diagram of triple quantum dots formed by applying voltages to L and P1 while keeping the double quantum dot state shown in Fig. 1c. We measure the $V_{\textrm{rf}}$ while varying $V_{\textrm{P2}}$ and $V_{\textrm{P3}}$ under the gate voltage conditions given in the figure caption. In Fig. 1d, the presence of three lines with different slopes corresponding to QD1, QD2, and QD3 confirms the formation of triple quantum dots. Additionally, similar to the charge stability diagram of the double quantum dots discussed earlier, the disappearance of charge transition lines over a wide range in the lower left region indicates that no electrons are present in each quantum dot in this area. In the upper-right region, the interdot coupling may become stronger and/or additional dots may appear, making it difficult to identify the exact number of quantum dots. However, in the few-electron regime, we confirm that the stability diagram clearly shows the formation of a triple quantum dot.

**Fig. 2:**
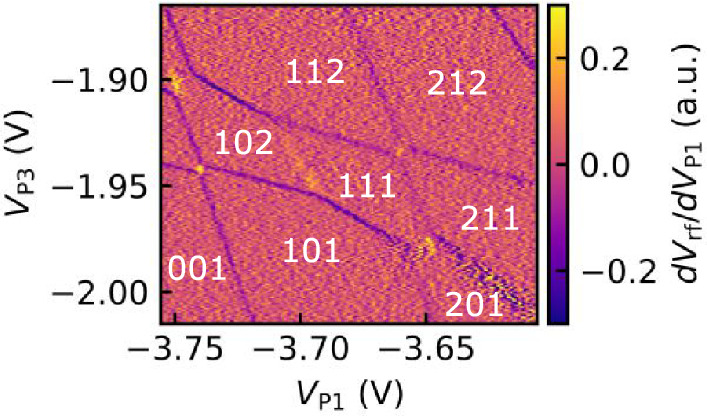
An enlarged view around the (111) regime in the charge stability diagram of the triple quantum dots in Fig. 1d. Gate voltage settings used for this measurement are $V_{\mathrm{{C}}} =-3.80$ V,$V_{\mathrm{{L}}} =-1.33$ V, $V_{\mathrm{{T1}}} = -1.71$ V, $V_{\mathrm{{T2}}} = -3.21$ V, $V_{\mathrm{{T3}}} = -3.60$ V, $V_{\mathrm{{P2}}} = -1.96$ V, $V_{\mathrm{{S2L}}}=-3.34$ V, $V_{\mathrm{{S2P}}} = -3.34$ V, and $V_{\mathrm{{S2R}}} = -3.38$ V.

**Fig. 3:**
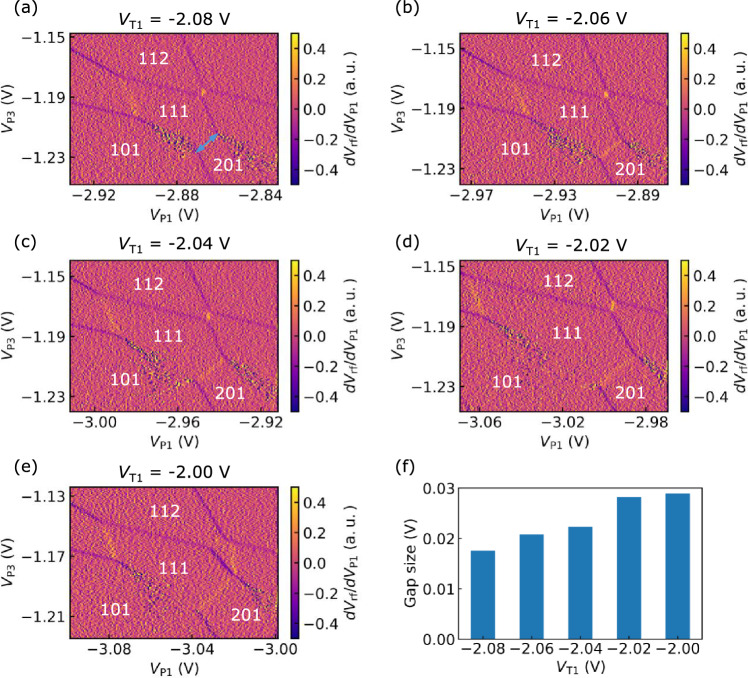
Tuning of the interdot coupling between QD1 and QD2; charge stability diagrams showing the voltage applied to T1 at different levels. (**a**) $V_{\mathrm{{T1}}} = -2.08$ V. The blue arrow indicates the gap size we analyzed. (**b**) $V_{\mathrm{{T1}}} = -2.06$ V. (**c**) $V_{\mathrm{{T1}}} = -2.04$ V. (**d**) $V_{\mathrm{{T1}}} = -2.02$ V. (**e**) $V_{\mathrm{{T1}}} = -2.00$ V. (**f**) The change in the size of the energy gap of level crossing in response to changes in T1.

Figure 2 shows an enlarged view of the charge stability diagram of the triple quantum dot around the state (111). For quantum bit applications, the electron state in (111), in which each dot contains a single electron, is important. In Fig. 2, $V_{\textrm{rf}}$ is measured while changing $V_{\textrm{P2}}$ and $V_{\textrm{P3}}$ under the voltage conditions listed in the figure caption. We can observe a few electron charge states with electron numbers (n$_{1}$ n$_{2}$ n$_{3}$) indicated in the figure. The gaps shown as the blue arrow in Fig. 3a indicates the presence of electrostatic coupling between the dots and depends on the capacitance between the corresponding quantum dots^49^. Therefore, the larger gap size suggests that the electrostatic coupling between QD1 and QD2, as well as between QD2 and QD3, is stronger than the coupling between QD1 and QD3.

Tuning of the interdot coupling is essential for controlling the electronic states and applications for qubits in the gate-defined multiple quantum dots. By varying the gate voltage of T1, we can manipulate the coupling between QD1 and QD2 as shown in Fig. 3a–e. The voltages of the other electrodes are adjusted to keep the charge state around the (111) state. Figure 3f shows the gap size at the crossing, which corresponds to the length of the charge transition line between (111) and (201), as a function of the gate voltage of T1. This figure indicates that increasing the T1 voltage enhances the gap at the level crossing between the electron configurations (111) and (201) due to increased electrostatic coupling between QD1 and QD2. Note that we demonstrate the control of capacitive coupling only between QD1 and QD2 in the present device. Our present ZnO device possesses a narrow operation window of gate voltage^22^. Moreover, each quantum dot is electrostatically coupled not only to the plunger gates P1, P2, and P3 but also to the tunnel gates T1 and T2. These factors make it challenging to realize a stable triple-dot configuration with tunable interdot coupling. To address this issue, further optimization of device parameters, such as gate voltages and device geometry, may enable controlled tuning of capacitive coupling on both sides as well as tunnel coupling. In our measurements, the observed changes in anticrossing gaps mainly reflect capacitive coupling controlled by the gate voltage. While this gap also depends on interdot tunneling, the absence of curvature at the crossing point of the transition lines suggests that the effect of interdot tunneling on the gap can be neglected^49^. A quantitative determination of interdot tunnel coupling, which can be evaluated by analyzing interdot transition widths^50^, will also be an important subject for future work.

**Fig. 4:**
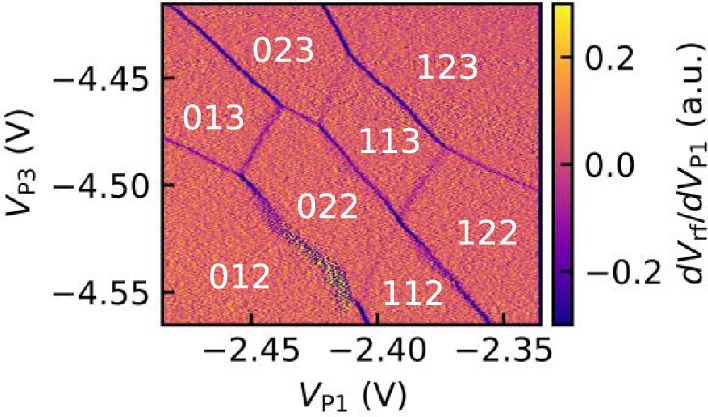
Charge stability diagram showing the QCA effect. Gate voltage settings used for this measurement are $V_{\mathrm{{C}}} =-3.50$ V, $V_{\mathrm{{L}}} = -1.63$ V, $V_{\mathrm{{T1}}} = -1.68$ V, $V_{\mathrm{{T2}}} = -2.93$ V, $V_{\mathrm{{T3}}} = -3.40$ V, and $V_{\mathrm{{P2}}} = -1.17$ V.

### Observation of the QCA effect

Having demonstrated the triple quantum dots operation, we are ready to observe characteristic phenomena that are observed only in triple or more quantum dots. We detect the tunneling phenomenon in which multiple electrons move simultaneously due to the Coulomb interaction QCA effect. Figure 4 shows the QCA effect observed in the charge stability diagram of triple quantum dots. In this measurement, we sweep $V_{\mathrm{{P1}}}$ and $V_{\mathrm{{P3}}}$ while fixing the other gate voltages as specified in the figure caption. These gate voltages are adjusted to cross the three kinds of charge transition lines corresponding to the three dots at a point and opening the gap. During rf reflectometry measurements, $V_{\mathrm{{S1L}}} = -2.35$ V, $V_{\mathrm{{S1P}}} = -1.72$ V are applied, and the QPC is used as a sensor in this measurement with a probe frequency of approximately 220 MHz. By identifying the electron configurations from the charge transition lines, regions corresponding to electron numbers (012), (111), (022), (112), (013), (113), (122), and (123) are labeled. In particular, the regions with electron configurations (022) and (113) are adjacent to each other. The transitions in this region require simultaneous movement of multiple electrons, and the QCA effect, which emerges only in more than three quantum dots, is observed in this region. The slope of the charge transition line between (022) and (113) is similar to that of QD2 (e.g., the transition line between (112) and (122)) because the electrostatic coupling between QD1 and QD2 is similar to that between QD2 and QD3. This condition is also observed in previous studies^45,51^. Note that the QCA stability diagram appears only in a small region compared to other regions, such as the one including state (123).

## Conclusion

In this study, we have formed multiple quantum dots in a zinc oxide heterostructure device and demonstrated few-electron states in triple quantum dots. We have shown that the interdot coupling can be systematically controlled by varying the gate voltage between quantum dots, confirming precise gate manipulability in ZnO quantum dot systems. Furthermore, we have observed the QCA effect, which cannot be observed in single or double quantum dots. This observation suggests that ZnO multiple quantum dots can also be utilized for wire-free cell networks and information transfer through QCA-based architectures. These achievements progress the utilization of the unique material properties of ZnO for quantum applications, while also providing an interesting platform for exploring fundamental quantum phenomena. The controllable multiple quantum dots in ZnO demonstrated in this study will open new possibilities for both scalable spin qubits and the investigation of fundamental physics in this promising material system.

## Methods

The heterostructure (Mg,Zn)O/ZnO is fabricated via molecular beam epitaxy on a Zn-polar ZnO (0001) substrate. The magnesium concentration within this heterostructure reaches approximately 2.5%. Gate insulator AlO$_\textrm{x}$ is deposited by atomic layer deposition. Ti/Au electrodes are deposited by electron-beam evaporation. Ohmic contacts are patterned by photolithography, while gate electrodes by electron-beam lithography, with both processes completed using standard lift-off techniques. The plunger gates are desined as a width of 20 nm with 100 nm pitches.

Hall effect measurements at 1.8 K reveal an electron density of $n = 4.9\times 10^{11}$ cm$^{-2}$ and mobility of $\mu = 170,000$ cm$^2$ V$^{-1}$ s$^{-1}$.

RF reflectometry measurements are performed by the demodulation circuit. Demodulated signal $V_\textrm{rf}$ is digitized for 125 MS/s sampling.

## Data Availability

The data that support the findings of this study are available in the article and upon reasonable request from the corresponding author.
